# Postintervention monitoring of peripheral arterial disease wound healing using dynamic vascular optical spectroscopy

**DOI:** 10.1117/1.JBO.27.12.125002

**Published:** 2022-12-24

**Authors:** Nisha Maheshwari, Alessandro Marone, Mirella Altoé, Stephen H.K. Kim, Danielle R. Bajakian, Andreas H. Hielscher

**Affiliations:** a New York University, Department of Biomedical Engineering, New York, United States; b Memorial Sloan Kettering Cancer Center, New York, United States; c Columbia University, Department of Radiology, New York, United States; d NY-Presbyterian/Columbia University, Department of Surgery, New York, United States

**Keywords:** peripheral arterial disease, dynamic optical spectroscopy, near-infrared spectroscopy, revascularization, wound healing

## Abstract

**Significance:**

Due to the persistence of chronic wounds, a second surgical intervention is often necessary for patients with peripheral arterial disease (PAD) within a year of the first intervention. The dynamic vascular optical spectroscopy system (DVOS) may assist physicians in determining patient prognosis only a month after the first surgical intervention.

**Aim:**

We aim to assess the DVOS utility in characterizing wound healing in PAD patients after endovascular intervention.

**Approach:**

The DVOS used near-infrared light (670<λ<850  nm) to record hemodynamic response to a cuff inflation in 14 PAD patients with lower limb ulcers immediately before, immediately after, and at a first follow-up 3 to 4 weeks after intervention. Ankle-brachial index (ABI) and arterial duplex ultrasound (A-DUS) measurements were obtained when possible.

**Results:**

The total hemoglobin plateau time differed significantly between patients with ulcers that reduced in size (N=9) and patients with ulcers that did not (N=5) 3 to 4 weeks after intervention (p value<0.001). Data correlated strongly (89% sensitivity, 100% specificity, and AUC=0.96) with long-term wound healing. ABI and A-DUS measurements were not statistically associated with wound healing.

**Conclusions:**

This pilot study demonstrates the potential of the DVOS to aid physicians in giving accurate long-term wound healing prognoses 1 month after intervention.

## 1 Introduction

It is estimated that 8 to 12 million people in the United States currently suffer from peripheral arterial disease (PAD).^1^ The disease is often comorbid with age, diabetes, and other kidney problems.^1^^–^^6^ PAD is caused by an accumulation of plaque in the vessels of the lower extremities, which leads to stenosis and reduction in blood flow. Early symptoms include claudication, numbness, or weakness and coldness in the lower leg or foot.^2^ Critical limb ischemia (CLI) is the most severe presentation of PAD and manifests as ulcers (chronic wounds), necrosis, or gangrene.^3^^–^^6^ Surgical intervention, such as balloon-only or stent angioplasties, is often necessary to treat patients with CLI. In patients with ulcers, a common way to determine the efficacy of intervention is to schedule patient follow-ups to visually inspect the wound and determine if the ulcer has healed or not. If an ulcer does not reduce in size after 12 weeks of standard care (i.e., infection management, changing bandages, etc.),^5^ a second intervention is typically prescribed.^6^ Unfortunately, a second intervention is required within 12 months for over 30% of patients due to the persistence of symptoms, including wounds.^7^^–^^9^

Visual examination of ulcer healing does not consider “invisible” underlying physiological changes that may occur long before any visible manifestations. Other methods have been developed to address this limitation in monitoring wound progression. One such monitoring method is the ankle-brachial index (ABI). The ABI determines the ratio of blood pressure measurements taken in the lower leg to those taken in the brachial artery. Wang et al. conducted a systematic review of observational studies that used the ABI to predict wound healing in diabetic foot ulcers. The data, taken from a total of 2376 patients, found the ABI to be a poor predictor of wound healing (sensitivity = 48% and specificity = 52%). Additionally, ABI measurements for patients with arterial calcification were found to be inaccurate across observational studies.^10^^–^^12^ Another widely used technique to monitor PAD treatment is arterial duplex ultrasound (A-DUS), which can detect vessel narrowing in the upper thigh. However, this approach does not provide information about the oxygen saturation levels of tissue in the foot and has difficulty visualizing small blood vessels.^13^^,^^14^ Neither the ABI nor A-DUS provides information about the distal perfusion in the foot, where ulcers most commonly occur in PAD patients.^13^^–^^15^ Despite these limitations, many physicians use the ABI to assist in PAD diagnosis and monitoring and employ A-DUS as a secondary monitoring method.^1^^,^^6^^,^^7^ Thus any new technology must be competitive with both the ABI and A-DUS in terms of diagnostic sensitivity.

New monitoring techniques have recently been developed to track treatment efficacy for arterial diseases. Cho et al. developed a machine learning model using data from electronic medical records that predicts chronic wound healing within 12 weeks (AUC = 0.717). The model uses demographic characteristics, patient clinical characteristics, and wound characteristics for prediction but does not account for interventions.^16^ Ruth et al. tested an implantable wireless sensor in a small animal model and successfully demonstrated the ability to monitor arterial occlusion. They found that continuous, long-term monitoring could detect the reappearance of PAD early and enable timely treatment of the disease. However, this method has yet to be tested in humans.^17^ Near-infrared imaging was used by Bochko et al. to analyze change over time in lower extremity ulcers. The imaging system performed well for segmentation and measurement of ulcers but was not tested for predictive capabilities.^18^

These current monitoring methods have limitations, such as inaccuracies in monitoring patients with arterial calcifications. There is a need for a technology that can predict wound healing outcomes as soon as possible after an intervention and with high accuracy across patient demographics. In a previous clinical pilot study, our group showed that near-infrared optical imaging has the potential to diagnose PAD in diabetic and nondiabetic patients due to differences in the hemodynamic responses of healthy versus PAD subjects to pressure cuff inflation.^19^ Therefore, we hypothesize that an optical method could monitor changes in wound healing dynamics within a PAD patient cohort. In this paper, we introduce a noninvasive, nonionizing, portable instrument—the dynamic vascular optical spectroscopy system (DVOS)—as a potential way to address the existing limitations with current monitoring techniques and aid in early prognoses of chronic wound healing.

## 2 Materials and Methods

### 2.1 Patient Study Population

We conducted a cross-sectional, observational pilot study at the Columbia University Irving Medical Center of New York City, New York, USA, between 2016 and 2019. The study complied with the declaration of Helsinki and was approved the by Columbia University Medical Center Institutional Review Board; informed written consent was obtained for all patients enrolled. The reporting guidelines followed for this paper are the STROBE reporting guidelines.^20^ Neither the patients nor the public were involved in the design, conduct, reporting, or dissemination of our research.

The nonrandom convenience sampling method was used to enroll patients diagnosed with PAD who were scheduled to undergo a surgical endovascular revascularization. Patients were scheduled for one of four possible interventions: balloon-only angioplasty (5 patients), stent angioplasty (5 patients), atherectomy (3 patients), or bypass surgery (1 patient). For both angioplasties, a catheter with a deflated balloon is inserted and guided to the artery of interest. The balloon is then inflated using water pressure, which presses plaque against the artery walls. The stent angioplasty also leaves an expanded stent in the artery to prevent restenosis.^7^^,^^9^ Atherectomy utilizes a high-speed blade at the end of the catheter, which is turned on and guided through the artery of interest to excise plaque on the artery walls.^8^ Bypass surgery connects arteries that no longer have continuous flow due to plaque build-up using either vein or synthetic grafts.^8^

This paper reports on 14 PAD patients who fulfilled the following criteria: presence of one or more ulcers on arteries of the lower leg or foot, availability of physician follow-up data ∼3 weeks after their intervention, and no prior interventions at least 6 months before the monitored intervention. The 6-month time point was chosen because the vasculature after both stent and balloon angioplasty, the two most common PAD interventions, is at risk of restenosis within 6 months after the intervention.^9^^,^^21^^,^^22^ Patient vasculature can be either patent or not patent within that timeframe. Taking measurements after this 6-month window increases the likelihood that the optical measurements accurately reflect the overall vascular health of the patient and not the results of the previous intervention. The characteristics of the patient study population are summarized in Table 1.

**Table 1:** Patient study population characteristics. Data are presented as n (%) or mean ± standard deviation.

Characteristics	Total
*Patient characteristics*	
Sex (male)	8 (57)
Age (years)	70.8 ± 17.9
BMI ($\mathrm{kg}/m^{2}$)	27.3 ± 4.6
Leg affected (left)	7 (50)
*Comorbidity*	
History of smoking (current or past)	6 (43)
Diabetic	10 (71)
Family history of diabetes	4 (29)
Hypertension	12 (86)
Hyperlipidemia	5 (36)
*Presenting symptoms*	
Claudication	6 (43)
Amputation	1 (7)
*Intervention*	
Balloon-only angioplasty	5 (36)
Angioplasty with stent	5 (36)
Atherectomy	3 (21)
Bypass	1 (7)

Patients were evaluated during physician follow-ups, when the size and location of the ulcer(s) were measured. Each patient had at least one follow-up 3 to 4 weeks after the intervention, and there was an average of two follow-ups per patient. The intervention outcome was determined using the ulcer measurement information from each patient’s last follow-up with the physician, which occurred between 3 weeks and 15 months after the intervention depending on the patient. The outcome was considered positive if the size of an ulcer was reduced or if the ulcer fully healed based on the physician’s visual examination. If the physician determined that an ulcer did not reduce in size or became larger, the outcome was considered negative. Of the 14 patients enrolled in this study, 9 had a positive outcome and 5 had a negative outcome.

### 2.2 Dynamic Vascular Optical Spectroscopy System Technology

The vasculature in the lower extremities of all patients was assessed with the so-called DVOS.

**Fig. 1:**
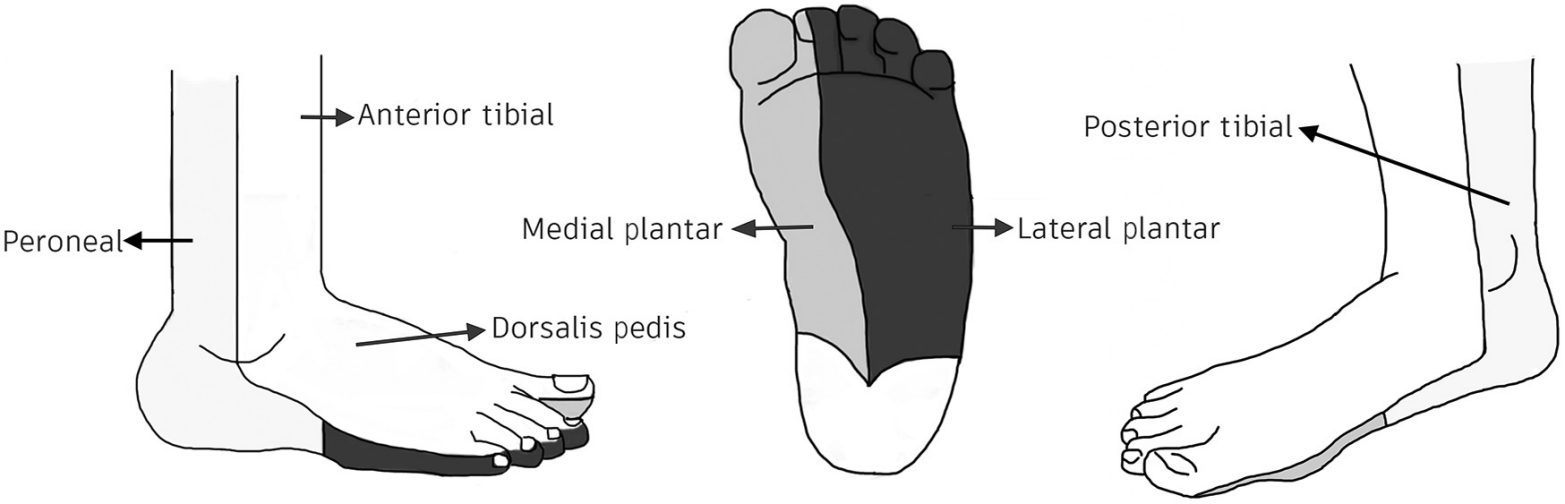
Map of angiosomes in the foot. Angiosomes of interest in this study include the peroneal, posterior tibial, anterior tibial, dorsalis pedis, lateral plantar, and medial plantar.

The DVOS consists of four patches that are placed at four different angiosomes of interest and simultaneously record tissue perfusion information in real time. An angiosome is a vascular territory that is characterized by and named after its principle feeding artery (Fig. 1).^23^

**Fig. 2:**
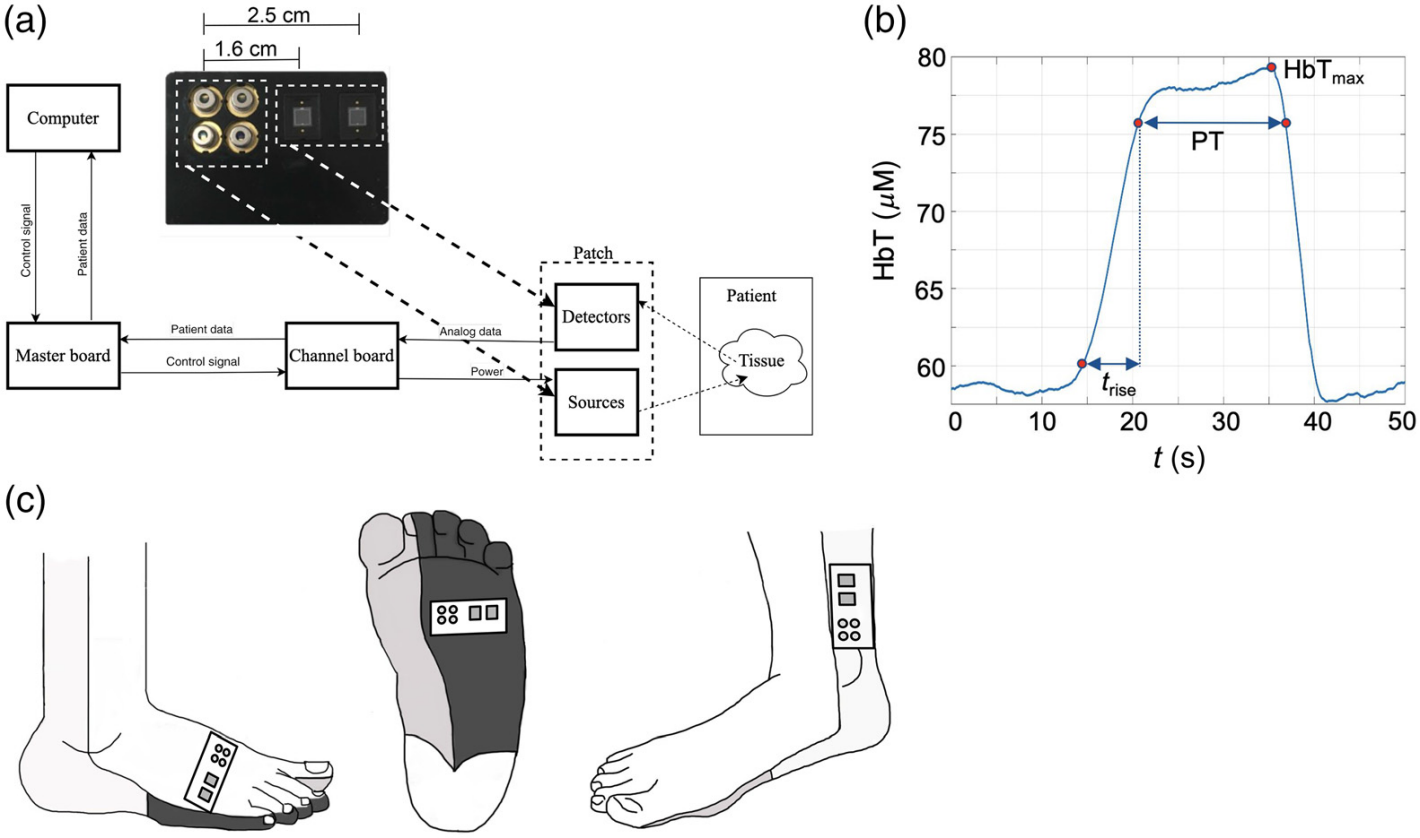
(a) Flowchart showing interaction between user device and DVOS with arrows showing direction of information transfers. The channel board ADC sends back patient data as a voltage reading. A close-up of a patch used for data acquisition is also shown. Source wavelengths clockwise from the top left are 850, 808, 670, and 780 nm. (b) Postoperative total hemoglobin (HbT) (μM) over time (s) from a patch on the medial plantar of a patient with a positive outcome. The PT, rise time ($t_{\text{rise}}$), and maximum HbT value (${\mathrm{HbT}}_{\max}$) are indicated on the curve. (c) Example patch placements and orientations for some of the angiosomes considered in this study (patches not to scale).

Each patch [Fig. 2(a)] has four 5.6-mm-diameter laser diodes that emit 5 mW of light at wavelengths of 780, 808, 670, and 850 nm (L780P010, L808P010, HL6748MG, L850P010, Thorlabs). These wavelengths were selected to provide a range of spectral information needed to reconstruct the oxygenated and deoxygenated hemoglobin concentrations in the underlying tissues. The light transmitted through the tissue is detected with two silicon photodiodes (S1337-33BR, Hamamatsu) located at distances of 1.6 and 2.5 cm from the laser diodes, respectively [Fig. 2(a)]. The laser diodes are driven by a 15-V laser diode driver (iC-WKN, iC Haus). Each wavelength is modulated in amplitude at a frequency of 5 kHz in a range from 0 to 3.3 V. The modulated signal is generated using a combination of a 1-kHz to 33-MHz oscillator (LTC6903, Linear Technology), a binary counter (M74HC4820, STMicroelectronics), and a SPI-controllable low-pass filter (LTC1569-6, Linear Technology) that is set to 72 kHz.

The DVOS is controlled with a laptop [Fig. 2(a)] that uses a MATLAB graphical user interface (GUI). Relevant clinical information can be extracted and displayed on the GUI in real time.

### 2.3 DVOS Data Acquisition

Data were acquired with the DVOS 1 to 3 h before the intervention (preintervention), 1 to 3 h after the intervention (postintervention), and at the first (∼3 to 4 weeks) follow-up (FU1) visit with the physician. Fourteen patients participated in the study, of which nine had a positive outcome and five had a negative outcome. During the preintervention visit, each patient had 1 to 4 patches placed on different angiosomes in the lower extremities. An example of patch orientation and position relative to the angiosomes can be seen in Fig. 2(c). Patches were secured with Velcro straps. Patch locations were determined for each individual patient based on the location(s) of their ulcer(s). Arteries on or nearest the ulcer were prioritized when choosing patch locations. For some patients, the ulcers were significantly large or painful enough that a patch could not be placed directly on the corresponding artery. In these cases, patches were placed on the preceding and following arteries (see Fig. 1) as applicable. All patches were placed in the same location across time points for each patient, allowing for comparison of the same arteries across time.

The data acquisition process consisted of five phases, each lasting ∼1  min. First, baseline values of hemoglobin concentration were acquired. Next, a thigh pressure cuff was inflated to 60 mmHg. The cuff took ∼10  s to inflate and was kept at this pressure for around 1 min. In the third step, the cuff was rapidly deflated, and data were subsequently acquired for 1 min. In the fourth phase, the thigh pressure cuff was inflated to 100 mmHg, which took around 10 s. The cuff was held at this pressure for around 1 min. Finally, the cuff was rapidly deflated, and data were collected for another minute following cuff deflation. The cuff inflations cause a venous occlusion while minimally (60 mmHg) or partially (100 mmHg) affecting the arteries. This allows blood to accumulate in the foot. The accumulation was recorded by the DVOS as an increment in absorbed light detected by the source–detector pair.

The data acquisition rate was the same for all patches and was determined by the number of patches simultaneously in use. When four patches were in use, all patches acquired data at 2.56 frames per second. Not all patients were able to accommodate four patches due to open wounds or bandages. In these cases, patches acquired data at either 3.41, 5.12, or 10.24 frames per second depending on whether three, two, or one patch(es) were used, respectively. The conversion to the time scale, which was used for all data analysis, was done using the data acquisition frame rate for each patient.

### 2.4 DVOS Feature Selection

Each of the DVOS source–detector pairs tracks changes in absorbed light and reports readings in real time as voltage measurements that are plotted on the MATLAB GUI. In a previous publications, we showed how the raw data collected by the DVOS are input to a diffusion-theory-based PDE-constrained multispectral reconstruction algorithm to extract relevant variables related to blood perfusion in the lower extremity arteries.^19^^,^^23^ These include the time it takes for blood to accumulate ($t_{\text{rise}}$) and the maximum total hemoglobin (${\mathrm{HbT}}_{\max}$) concentration in the artery [Fig. 2(b)].

In previous studies,^19^^,^^24^ we found that the plateau time (PT) has a strong correlation with vascular health, and the PT was therefore the only variable considered for this study. The PT is extracted from the reconstructed HbT concentration data and is the difference in time (s) between the two points on the HbT concentration curve corresponding to 90% of ${\mathrm{HbT}}_{\max}$ [Fig. 2(b)]. This is a metric for how long it takes blood to pool in the foot after the cuff inflation. The PT for a single patch is given as the mean PT of the minimal and partial cuff inflation trials per patient per time point.

Measurements were split into two groups based on the patch location. The general (Gen) group data are given as the mean PT data of all patches per patient per time point. As this is the mean of all patches, data are independent from ulcer location. The localized (Loc) group is PT data only from the patch on the artery nearest the ulcer on the arterial tree (Fig. 1) per patient per time point. The nearest ulcer is, in preferential order, either the patch on the angiosome corresponding to the ulcer, the mean of the patch(es) below the affected artery, or the patch directly above the affected artery on the arterial tree.

Data were also aggregated into three features based on differences in the PT between each time point as explained in Table 2. Data in the PostPre group are the difference in the PT assessed at the post and preintervention time points. Data in the FU1Pre group are the difference in the PT assessed at the FU1 and preintervention time points. Data in the FU1Post group are the difference in the PT assessed at the FU1 and postintervention time points.

**Table 2:** Features identified for optical, ABI, and A-DUS imaging modalities grouped by patch location.

Method	Group	Feature	Description
Optical (DVOS)	Gen	PostPreGen	Difference in PT in seconds between post and preintervention time points, where PT is from the mean of all patches for each patient
		FU1PreGen	Difference in PT in seconds between FU1 and preintervention time points, where PT is from the mean of all patches for each patient
		FU1PostGen	Difference in PT in seconds between FU1 and postintervention time points, where PT is from the mean of all patches for each patient
	Loc	PostPreLoc	Difference in PT in seconds between post and preintervention time points, where PT is from the patch nearest the ulcer for each patient
		FU1PreLoc	Difference in PT in seconds between FU1 and preintervention time points, where PT is from the patch nearest the ulcer for each patient
		FU1PostLoc	Difference in PT in seconds between FU1 and postintervention time points, where PT is from the patch nearest the ulcer for each patient
ABI and A-DUS	Gen	PreGen	Mean pressure (ABI) or PSV (A-DUS) ratio of the arteries of interest at the preintervention time point for each patient
		FU1Gen	Mean pressure (ABI) or PSV (A-DUS) ratio of the arteries of interest at the FU1 time point for each patient
	Loc	PreLoc	Pressure (ABI) or PSV (A-DUS) ratio of the artery nearest the ulcer at the preintervention time point for each patient
		FU1Loc	Pressure (ABI) or PSV (A-DUS) ratio of the artery nearest the ulcer at the FU1 time point for each patient

Data from the Gen and Loc groups were found to be highly correlated for the same differences in time points (R>0.75). Of the six features identified above, this paper focuses on the set of three independent features associated with the Gen group: PostPreGen, FU1PreGen, and FU1PostGen. The Loc group varied by patient in terms of which location was considered “local” to the affected artery (i.e., on, above, or below it). Thus the Gen group was chosen for analysis as a more reproducible and standardizable metric.

### 2.5 ABI and A-DUS Data Acquisition

In addition to the DVOS data, ABI and A-DUS data were available for some patients at both the preintervention and FU1 physician visits.

There were five patients with preintervention ABI data, with positive (N=3) or negative (N=2) patient outcomes. There were 10 patients with FU1 ABI data, with positive (N=7) or negative (N=3) patient outcomes. Data are considered only for individual time points (as opposed to the difference across time points) as is standard for ABI measurements.

These data were taken using the standard medical procedures for ABI measurements.^24^^–^^26^ Arteries of interest measured for this study were the posterior tibial and dorsalis pedis. The ABI of each artery was recorded as the ratio of systolic blood pressure at the artery of interest to that at the brachial artery. ABI data considered are the pressure ratio measurements from the artery closest to the ulcer location and the mean pressure ratio measurements from the two arteries.

There were nine patients with available preintervention A-DUS data, with positive (N=7) or negative (N=2) patient outcomes. Additionally, there were 11 patients with FU1 A-DUS data, with positive (N=7) or negative (N=4) patient final outcomes. Data are considered only for individual time points (as opposed to the difference across time points) as is standard for ultrasound measurements.

These data were taken using the standard medical procedures for A-DUS measurements.^27^^–^^29^ Arteries of interest measured for this study were the peroneal, posterior tibial, and anterior tibial. The peak systolic velocity (PSV) ratio was recorded as the ratio of PSV in the artery of interest to the PSV in the common femoral artery. A-DUS data considered are the PSV ratio measurements from the artery closest to the ulcer location and the mean PSV ratio measurements from the three arteries.

Both ABI and A-DUS data each have four features of interest based on the patch location and time of data acquisition as described in Table 2. As explained in Sec. 2.4, the patch location was designated Gen or Loc, and the time points were designated as Pre and FU1. There was no correlation between any of the features of interest for the ABI data or the A-DUS data.

### 2.6 Statistical Analyses

To identify the independent optical, ABI, and A-DUS features, we performed a Spearman’s rank-order correlation to determine if there were linear dependencies between any pair of parameters. A coefficient of R>0.75 was considered highly correlated. An independent-samples t-test was performed comparing the PTs (optical data) between patients with positive and negative outcomes. The data were reported as the mean ± standard deviation. The test statistics were adjusted with the Bonferroni correction. We also investigated the difference in pressure ratios (ABI data) and PSV ratios (A-DUS data) between the patients with positive and negative outcomes. The statistical analyses were performed using RStudio software (RStudio release 1.4.1717; RStudio, Boston, MA, USA). Feature classification performances for optical, ABI, and A-DUS features were determined using receiver operator characteristic (ROC) curves. The ROC curves and associated AUCs were generated using MATLAB software (MATLAB 2020; MathWorks Inc., Natick, MA, USA).

## 3 Results

### 3.1 DVOS Characteristics

**Fig. 3:**
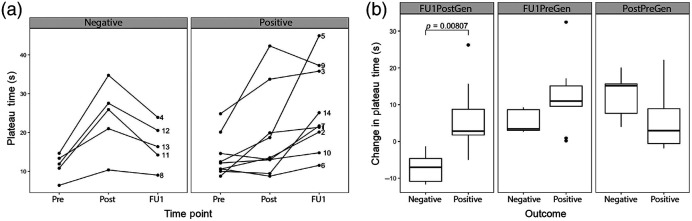
(a) PT in seconds for patients with a positive (N=9) and negative (N=5) outcome at the preintervention (Pre), postintervention (Post), and first follow-up (FU1) time points. (b) Change in PT in seconds between FU1 and Post (FU1PostGen), FU1 and Pre (FU1PreGen), and Post and Pre (PostPreGen).

The PT trends between the preintervention, postintervention, and FU1 time points were observed for patients with positive and negative wound healing [Fig. 3(a)]. The PT increased from pre to postintervention for patients in both groups. Patients in both groups reached HbT saturation during the pressure cuff inflation at the postintervention time point. The PT values had a more pronounced increase in patients with a negative outcome. The trend from postintervention to FU1 differed across groups. All patients with negative outcomes had a decrease in the PT from postintervention to FU1 and followed the same PT trend over the three time points. Most patients with negative outcomes did not reach HbT saturation at the FU1 time point. All but one patient with positive outcomes had an increase in PT from postintervention to FU1, with more variation in the trend lines between the three time points than is seen in the negative outcome patient group [Fig. 3(a)]. These patients achieved saturation at both the postintervention and FU1 time points.

### 3.2 DVOS Classification

Of the three independent features identified for the DVOS data, only FU1PostGen had a statistically significant difference (t=−3.78, 11.8 df, P=0.008) between patients with positive (N=9) and negative (N=5) outcomes [Fig. 3(b)]. Patients with a positive outcome had a change in PT=6.7±9.3  s, whereas patients with a negative outcome had a change in PT=−7.1±4.3  s. There is more variation in the PT difference from postintervention to FU1 among patients with positive outcomes.

**Fig. 4:**
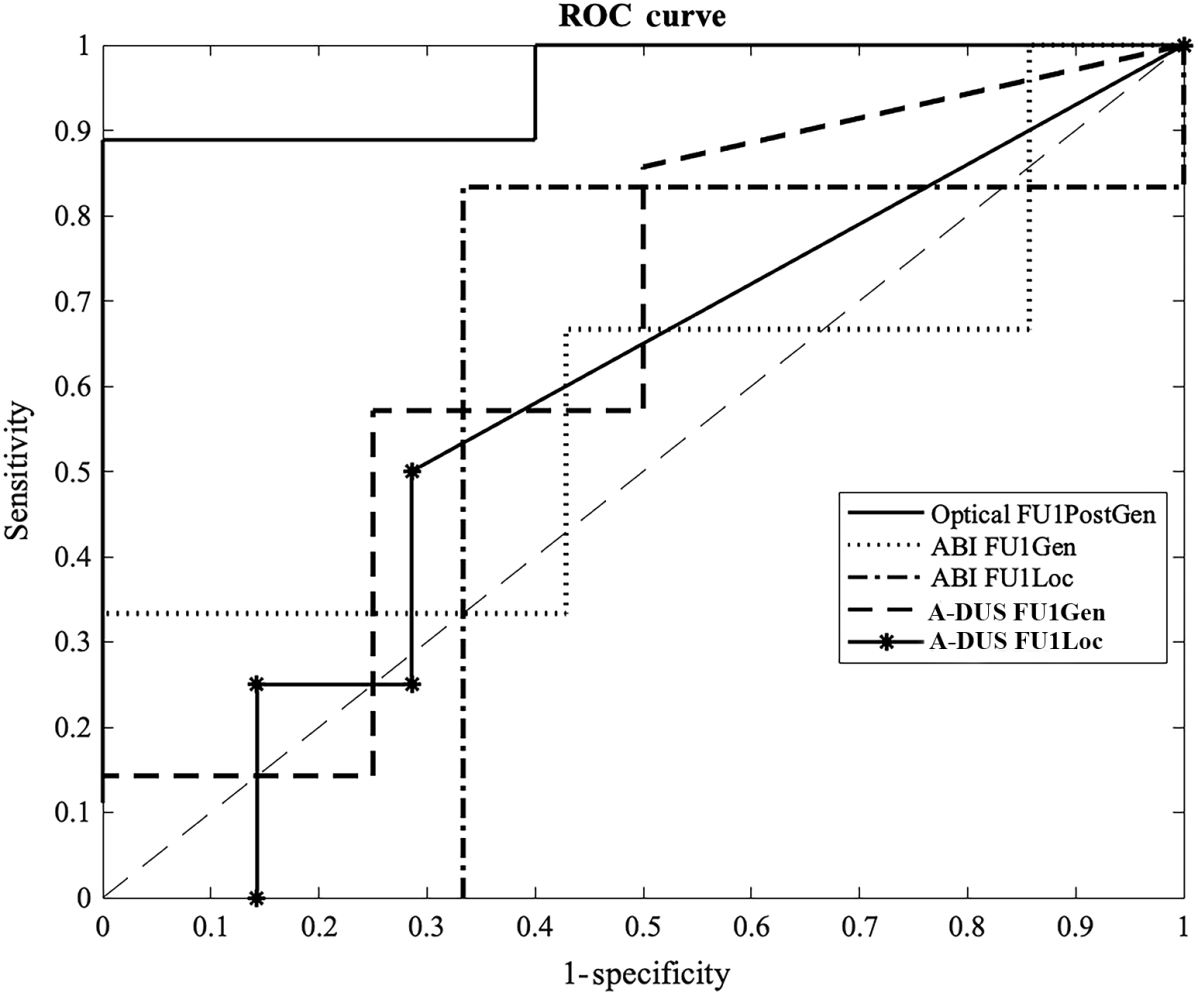
ROC curves for optical, ABI, and A-DUS (AD) data at the first follow-up (FU1). Areas under the curve are 0.96, 0.57, 0.56, 0.64, and 0.57 for Optical FU1PostGen, ABI FU1Gen, ABI FU1Loc, AD FU1Gen, and AD FU1Loc, respectively.

We determined the association between FU1PostGen and patient outcome based on a ROC curve (Fig. 4). The FU1PostGen data had a strong association with wound healing; classification of patients considering only this feature performed very well with a sensitivity of $S_{e}=89\%$, specificity of $S_{p}=100\%$, positive predictive value (PPV) of 100%, and negative predictive value (NPV) of 83% (AUC=0.96). Based on the optimal cutoff of the ROC curve, a patient was likely to worsen when the difference between FU1 and postintervention PTs was below −1.3  s. The optimal cutoff value for the FU1PostGen data was found using the Youden index of the ROC. The Youden index J is the sum of sensitivity and specificity-1. The sensitivity–specificity pair that is closest to (0, 1) on the ROC curve yields the largest J,^30^ and we determined the cutoff value at this point. At this cutoff, only one patient was misclassified—a patient with a positive outcome classified as having a negative outcome.

### 3.3 ABI and A-DUS Characteristics

Neither the four ABI features nor the four A-DUS features were found to have a statistically significant difference between patients with positive and negative wound healing outcomes. Groups did not differ across time points nor patch locations.

Both the ABI and A-DUS data at the FU1 time point had weak associations with wound healing; classifications performed poorly with either high sensitivity or specificity, not both. Of the four features (ABI FU1Gen/Loc, A-DUS FU1Gen/Loc), A-DUS FU1Gen had the highest potential with a $S_{e}=86\%$ and $S_{p}=50\%$ (AUC=0.64; Fig. 4).

### 3.4 Comparison of DVOS, ABI, and A-DUS

In addition, we compared the performance of the DVOS with both the ABI and A-DUS systems in determining patient clinical outcome (Table 3) by the time of the first follow-up visit (FU1). For this comparison, we classified patients based on cutoff values associated with predicting patient outcome for each modality. For ABI data, we used a cutoff value of 0.9, which is the threshold most used to diagnose PAD in the current literature.^9^^,^^21^^,^^22^ Similarly, we used the most cited cutoff value for PAD diagnosis with A-DUS, which is a PSV ratio of 2.4.^14^^,^^27^^,^^29^ The cutoff for DVOS data was the difference in postintervention and FU1 PT of −1.3  s. Section 3.2 details how the DVOS cutoff value was determined.

**Table 3:** Truth table comparing patients’ clinical outcomes with the assigned outcomes by optical, ABI, or A-DUS data acquisition.

Patient	CO^a^	OO	ABFG	ABFL	ADFG	ADFL	CO = OO	CO = ABFG	CO = ABFL	CO = ADFG	CO = ADFL
1	1	1	1	1	1	1	**1** ^c^	**1**	**1**	**1**	**1**
2	1	1	1	0	1	1	**1**	**1**	0	**1**	**1**
3	1	1	0	0	1	0	**1**	0	0	**1**	0
4	0	0	0	0	X	X	**1**	**1**	**1**	X	X
5	1	1	X^b^	X	X	X	**1**	X	X	X	X
6	1	1	0	0	1	1	**1**	0	0	**1**	**1**
7	1	1	0	0	X	X	**1**	0	0	X	X
8	0	0	0	0	1	1	**1**	**1**	**1**	0	0
9	1	0	1	1	1	1	0	**1**	**1**	**1**	**1**
10	1	1	X	X	1	1	**1**	X	X	**1**	**1**
11	0	0	X	X	1	1	**1**	X	X	0	0
12	0	0	1	1	1	1	**1**	0	0	0	0
13	0	0	X	X	1	1	**1**	X	X	0	0
14	1	1	0	X	1	1	**1**	0	X	**1**	**1**

a CO, clinical outcome; OO, optical outcome; ABFG, ABI FU1Gen; ABFL, ABI FU1Loc; ADFG, A-DUS FU1Gen; and ADFL, A-DUS FU1Loc.

b An “X” indicates insufficient data.

c Bold indicates that the measured outcome matches the clinical outcome.

Only one patient was incorrectly classified by the DVOS with the optimal cutoff value. This was the only patient who had a positive clinical outcome in which the PT value decreased from the postoperative to the FU1 time points. Both the Gen and Loc data from ABI and A-DUS correctly classified this patient. There are many discrepancies in patient classifications between the ABI and A-DUS data and between the Gen and Loc groups of both the ABI and A-DUS data as seen in Table 3.

## 4 Discussion

Many patients in later stages of PAD suffer from ulcers in the lower extremities.^3^^–^^6^ Due to clogging of the arteries, patients do not have the healthy blood perfusion necessary for proper wound healing.^31^^–^^33^ Minimally invasive revascularization is becoming an increasingly popular treatment method for PAD patients, with the goal of restoring normal blood flow to the lower leg and foot.^7^^–^^9^ Early determination of the efficacy of treatment is still lacking due to limitations in the current technology. Optical methods offer a potential way to address these limitations, as information on the hemodynamics of specific arteries of interest can be monitored over time for patient-specific vasculature.

We expect that patients with positive wound healing outcomes will have different blood perfusion characteristics than patients with negative outcomes.^31^^–^^33^ Specifically, blood pooling in arteries of interest will differ depending on the amount of clogging in that artery. Patients with healthy arteries should reach saturation in HbT faster than patients with unhealthy arteries. The PT is one metric used to quantify the amount of time that blood stays at saturation (or if saturation is reached at all) and consequently the healthiness of the arteries. The data in this pilot study confirm that the PT may be a good classifier for wound healing in PAD patients and outperforms existing noninvasive monitoring techniques.

All patients, including those with negative outcomes, show improvement in the PT immediately after their intervention (i.e., PostPre), which indicates that a partial or complete opening of the artery was achieved from the intervention. Patients with a negative outcome had larger PostPre values than patients with a positive long-term outcome. This may indicate that there was a difference in relative vascular health in the two groups before intervention. At FU1, almost the entire patient cohort improved from the preintervention data, due to low PT values at the preintervention [Fig. 3(a)]. This indicates both that all patients started out with unhealthy vasculature and that the interventions affected blood perfusion in patients up to 1 month afterward. FU1Post is the most reliable change in the PT likely because it does not include preintervention data, which is low for all patients. Neither the ABI nor A-DUS were able to distinguish between patients with positive and negative wound healing outcomes by the FU1 time point. This is true for both the Gen and Loc groups at the FU1 time point. The general data marginally outperforms the local data (Table 3).

Changes in the PT between the postintervention and FU1 time points (FU1Post) are statistically significant between patients with positive and negative outcomes. These differences are seen regardless of patch location; both the Gen and Loc groups have statistically significant differences (P<0.01) between patient outcomes and are highly correlated (R>0.75) across all time points. The FU1PostGen feature derived from DVOS data correlated strongly (R>0.75) with wound healing. Using a cutoff value of change in PT=−1.3  s results in a $S_{e}=89\%$, $S_{p}=100\%$, PPV of 100%, and NPV of 83%, outperforming the ABI and A-DUS modalities. The demographic diversity within the patient cohort indicates that DVOS data correlates strongly with wound healing for patients across varying ages, genders, etc. The results suggest that DVOS has the potential to be used by physicians to monitor wound healing and determine a patient’s prognosis by the 3-week follow-up, which is sooner than some existing technologies can. The DVOS may also address the limitations of the ABI and A-DUS.

These preliminary results show that the DVOS has high potential in predicting wound healing outcome after a surgical intervention for PAD patients. However, the study has low statistical power due to the small patient cohort, and a larger cohort is therefore needed to conclusively support these findings. Based on the sampled standard deviations of the patients with positive (change in PT=6.7±9.3  s) and negative (change in PT=−7.1±4.3  s) outcomes, we estimate that, for a desired 95% confidence interval with half width equal to one standard deviation of the difference in sample means (α=0.05, β=0.04), a clinical study with at least 56 patients should be performed.^34^

Patient follow-up retention was a further limitation of this study, as the time between the intervention and the last follow-up varied on a patient specific basis. Long-term outcome was determined using the ulcer measurement data from the last physician follow-up, and patient progression was not tracked after this follow-up. The long-term outcome may be different from the reported outcome if the wound progression changed significantly after the last measurement.

The goal of this pilot study was to assess the potential of the DVOS to classify wound outcome for a subset of PAD patients with ulcers and in need of surgical intervention, regardless of other demographic information. Based on the results of this pilot study, we plan to enroll a larger patient cohort, which would allow for comparisons based on variations such as race, age, and gender. We plan to investigate the classification abilities of the DVOS for patients within specific groups to confirm system efficacy across different demographics. Monitoring of patient activity between follow-up visits may be a parameter to consider in the future that could enhance the classification ability outlined in this paper.

Our system uses four distinct wavelengths, making it possible to find both the scattering and ${\mathrm{SO}}_{2}$ information from the DVOS data. In future research with a larger patient cohort, we aim to add this information to further identify differences across wound healing groups. This may provide physicians with further markers to assist in making a prognosis.

## Code, Data, and Materials Availability

The datasets presented can be made available for noncommercial use upon reasonable request to the author, after acceptable completion of the university’s data use agreement. Requests to access the datasets should be directed to ndm9911@nyu.edu.
